# First line antiretroviral treatment failure and associated factors among people living with HIV in northwest Ethiopia

**DOI:** 10.4314/ahs.v21i1.34

**Published:** 2021-03

**Authors:** Andualem Genet, Zewdie Mekonnen, Endalew Yizengaw, Daniel Mekonnen

**Affiliations:** 1 Department of Medical Microbiology Immunology and Parasitology, College of Medicine and Health Sciences, Bahir Dar University, Ethiopia; 2 Department of Medical Biochemistry, College of Medicine and Health Science, Bahir Dar University, Bahir Dar, Ethiopia; 3 Biotechnology Research Institute, Bahir Dar University, Bahir Dar, Ethiopia

**Keywords:** HIV, viral load, treatment failure, Ethiopia

## Abstract

**Background:**

Anti-retroviral treatment enhances the immune status and reduces unwanted outcomes. However, development of treatment failure and drug resistance raises concern over lifelong treatments to chronic diseases such as HIV/AIDS.

**Objectives:**

This study determined proportion of treatment failure (TF) and identified factors associated with TF among people living with human immunodeficiency virus (HIV) in Bahir Dar, Northwest Ethiopia.

**Methods:**

Facility based cross sectional study was carried out from November, 2017 to April, 2018. Sociodemographic and clinical data were collected using structured questioner. Blood sample was collected and analyzed for viral load, complete blood count (CBC), liver and kidney function test and CD4 count. A patient is declared as treatment failure when viral load value is higher than 1000 RNA copies/ml in two consecutive viral load analyses within three months interval. Data were entered and analyzed using SPSS version 23. To identify factors associated with TF, logistic regressions model was employed.

**Results:**

A total of 430 people who had six months and above antiretroviral treatment (ART) follow up were enrolled in the study. Of these, 57.9% were females & the mean age was 38 years. The mean month of ART follow up was 83 months. In the first viral load analysis, 106 (24.7%) of the cohort were virologically failed. These failed people were followed for 3 months with intensive adherence support; then second viral load analysis showed a viralogical failure among 65 people of the second cohort. Thus, the overall viralogical failure or TF was 15.1%. The re-suppression rates were 41(38.7%). Male gender, people with history of drug discontinuation, poor adherence, irregular time of drug intake, multiple sexual practice showed significant association with TF. Moreover, base line and current CD4 counts of <200 cells/ml also demonstrated significant association with TF.

**Conclusion:**

Significant proportion of treatment failure was reported in the present study. Moreover, behavioral factors such as drug discontinuation, poor adherence, multiple sexual partner were associated with treatment failure. Hence, to avoid TF, regular patient counseling and monitoring should be in place. To identify the predictors for treatment failure, further follow-up study is desirable.

## Background

Human immunodeficiency virus (HIV) is a cause of acquired immune deficiency syndrome (AIDS). Based on World Health Organization (WHO) report of 2015, 36.7 million people were living with HIV. In the same year, the disease claimed nearly 2.3 million new infections and 1.6 million death, respectively 1. Sub-Saharan Africa accounted 69% of the global HIV burden^2^. The prevalence of HIV in Ethiopia was 1.16 %. However, this figure varied across regional states. For instance, HIV prevalence was lowest (0.54 %) in Southern Nation Nationality and Peoples state (SNNPS) and highest (4.9%) in Gambella regional state 3. Over 94% of HIV-1 infection in Ethiopia is dominated by two phylogenetically distinct subtype C clades, the Ethiopian (C'-ET) and East African (C-EA) clades followed by Ethiopian South Africa clade (E-SA) 4.

First-line ART regimen is a cocktail of two nucleoside reverse-transcriptase inhibitors (NRTIs) plus a non-nucleoside reverse-transcriptase inhibitor (NNRTI). Of these, tenofovir disoproxil fumarate (TDF) with 3TC or emtricitabine (FTC) and efavirenz (EFV) as a fixeddose combination is recommended as the preferred option to initiate ART^5^.

While the rapid development of sensitive and specific point of care diagnostic technique is one of the success stories in the history of HIV; development of curative treatment and vaccine could not be a reality. Recently, the clustered regularly interspaced short palindromic repeat (CRISPR)/CRISPR-associated nuclease 9 (Cas9) system has been engineered as an effective gene-editing technology with the potential to treat HIV-1/AIDS. It can be used to target cellular co-factors or HIV-1 genome to reduce HIV-1 infection and clear the provirus, as well as to induce transcriptional activation of latent virus in latent viral reservoirs for elimination 6.

The sustainable development goal (SDG) for the year 2020 set a target known as 90-90-90. This is to test, to have sustained ART and to achieve viral suppressions rate of 90% of the eligible people 7. Treatment failure (TF), defined as the progression of disease and replication of HIV despite of medication. Treatment failure can be monitored virologically (Plasma viral load above 1000 copies/ ml based on two consecutive viral load measurements after 3 months, with adherence support) and immunologically (a fall in CD4 cell count to baseline (or below) or a 50% reduction from on treatment peak value or presence of persistent CD4 cell count below 100 cells/mm^3^) and clinically (the occurrence of new or recurrent WHO stage 4 or some stage 3 conditions^8^. Of these, virological failure is considered sensitive, specific and gold standard method for monitoring TF^9^, ^10^. Recently, viral load test-based treatment monitoring was introduced in Ethiopia 3.

Based on nationwide survey of Ethiopia, the prevalence of transmitted drug resistance (TDR) was 3.9%^11^. Based on immunological criteria, the prevalence of TF was 4.1% in Gondar 12 and 21% in Debre Markos 13, 5.3% in Jimma 14 and 31.2% in central Ethiopia, Addis Ababa 15. Based on single plasma viral load (PVL) study, prevalence of TF was 10.7% in Bahir Dar, Ethiopia 16. Based on a study in Gondar immunological failure and virologic failure were 13.2 and 14.7%, respectively 17. From 18 articles reviewed; the pooled proportion of first line treatment failure among ART users in Ethiopia was 15.3%. The prevalence of virologic treatment failure was gauged at 5.8% 18.

Several lines of evidences showed that, poor adherence^12^ ^18^ use of sub-optimal drug combinations 19 staying for long period of time on the same regimen and dependency 20, 21 were the explaining factor for TF. Moreover, first-line antiretroviral treatment failure were associated with discontinuation of ART, baseline CD4 lymphocyte count ≤50 cells/mm^3^ 22. not disclosed, advanced WHO clinical stage III/IV, change in regimen and being co-infected were statistically significant factors for treatment failure 18. A recent institution-based retrospective follow-up study among 402 children in Amhara regional hospitals were conducted. Treatment failures rate within the follow-up period were 12.19% (95% CI: 8.5, 15.88). This study also found that the overall incidence density rate was 3.77% per 100 person-years observation. Virological failure accounts 48.98% followed by immunologic (28.57%) and mixed failures (22.44%). Poor ART adherence, drug regimens, AZT-3TC-NVP, and AZT-3TC-EFV, children whose both parents were died and WHO clinical stage-4 were found to be predictors for TF among the included children 23

Periodic surveillance is required for monitoring the prevalence of TF. Hence, the aim of this study was to determine the prevalence of first-line ART TF and identify factors associated with TF. This study also calculatedthe added value of second viral load on avoiding premature switching to the second line treatment.

## Materials and Methods

### Study design, Period and setting

A facility based cross sectional study was conducted from November, 2017 to April, 2018 at three ART sites in Bahir Dar City administration namely; Felege Hiwot Referral Hospital (FHRH), Han Health Center and Adinas General Hospital. More than 6300, 2000 and 200 patients were on ART at the three health facilities, respectively.

### Study population

All people living with HIV (PLWHIV) in Bahir Dar city administration were the source population and those who attended ART follow up at the three health facilities during the study period were the study population. PLWHIV who had ART follow-up period of at least 6 months were included. Only those on first line ART were included.

### Sample size and sampling technique

Sample size was determined using single population proportion formula

$$\begin{array}{c} \text{n=z2 x p (p-1)}\\ \text{D2} \end{array}$$

Where, n= sample size; p=prevalence taking 31.2% prevalence of TF based viral load test in Addis Ababa 15; z=1.96= statistic level of confidence; D = margin of error taking 5%; adding 10% non-response rate finally reached to 430. Sample size was proportionally allocated to the three study sites. Patients who gave written consent and ascent were consecutively enrolled until the required sample size obtained.

### Study variables

Virological treatment Failure (VTF) or simply TFwas the dependent variable.

### Data collection

Sociodemographic and baseline clinical data were captured by ART nurses using a pretested questionnaire. Baseline CD4 value, WHO stage, months of ART follow up and baseline regimens were extracted from ART registration log book.

### Specimen collection and transportation

Blood sample was collected in duplicate using vacutainer sample collection apparatus from each participant. For serum glutamic pyruvic transaminase (SGPT), serum glutamic oxaloacetic transaminase (SGOT) and creatinine analysis, 5 ml of blood was collected with serum separator tube containing gel. For plasma viral load (PVL), CD4 and complete blood count (CBC) tests, 4 ml of whole blood was collected with K3 EDTA anticoagulant coated vacuum test tube^24^. Samples were shipped to the Amhara Public Health Institute (APHI) using cold chain system.

### Plasma viral load analysis

The RNA was extracted from 200 µl of plasma sample using Abbott m2000sp automated equipment. Then 50 µl of RNA sample-eluate and 50 µl master mix (HIV-1 oligonucleotide reagents, activator, rTth polymerase) have been combined and run for amplification. The target sequence for HIV-1 is in the pol Integrase region of the HIV-1 genome. The target RNA region converted to cDNA by the reverse transcriptase activity of the thermostable rTth DNA polymerase.

Briefly, the HIV-1 and internal control (IC) reverse primers anneal to their respective targets at 59oc for 30 min for reveres transcription. The optimum denaturation, annealing and extension temperature and time were at 92°c for 30 second, 56°c 20 second respectively. Hybridization and detection take place at 35°c. The amount of HIV-1 target sequence is measured through the use of fluorescent-labeled oligonucleotide probes. The amplification cycle at which fluorescent signal detected by the Abbott m2000rt is proportional to the log of the HIV-1 RNA concentration present in the original sample^24^. The detection limit of Abbott m2000sp is 150 RNA copies/ml^25^. A patient is declared as treatment failure when viral load value is higher than 1000 RNA copies/ml in two consecutive viral load analyses within three months interval.

### Data analysis and quality

The data were entered and scrutinized using SPSS (Statistical package for social sciences IBM SPSS 23). The information was summarized using frequency, percentage and mean. Moreover, logistic regression was computed to identify factors associated with TF. P-value of ≤ 0.05 considered statistically significant.

The questionnaires were pre-tested and validated. Internal quality controls (IQC) and calibration have been done at every reagent lot changed. Viral load Abbott M2000 machine was monitored with negative, low positive and high positive controls at every session run of viral load.

## Results

### Demographic and clinical characteristics

A total of 430 PLWHIV and who took first-line ART drugs for six and more months were recruited. Of these, 249 (57.9%) were female, 396 (92.1%) were urban dwellers. The mean age of participants was 38 years (ranges 12–67 years). Table 1 below summarizes the socio-demographic variables.

**Table 1:** Socio demographic characteristics of participants Bahir Dar, Nov, 2017–April, 2018

Variable	Category	Frequency(N)	Percent (%)
Gender	Female	249	57.9
	Male	181	42.1
Address	Urban	396	92.1
	Rural	34	7.9
Education	Illiterate	125	29.1
	1–8 grade	128	29.8
	9–12th grade	103	24.0
	12plus	74	17.2
Marital status	Married	196	45.6
	Single	71	16.5
	Divorced	93	21.6
	Widowed	70	16.3
Age	< 15	3	0.7
	16–25	26	6.0
	26–35	123	28.6
	36–45	163	37.9
	46–55	88	20.5
	>55	27	6.3
Occupations	Daily laborer	97	22.6
	Self-business	142	33.0
	Governmental employee	93	21.6
	Farmer	24	5.6
	Student	12	2.8
	No work	21	4.9
	Others	41	9.5
Body Mass Index (BMI)	<15.5	95	22.2
	18.6–25.5	284	66.0
	25.6–30.0	47	10.9
	>30.1	4	0.9
Total		430	100

Of the total 430 participants, 44(10.2%) and 119(27.7%) had current and previous history of tuberculosis (TB), respectively. Diabetes mellitus (DM) was reported from 10 (2.3%) of the participants. Ninety-four percent of the patients did not have history of dependency, 116(27%) had history of multiple sexual partner, 123(28.6 %) used condom regularly. The average length of stay on ART was 83 months (Range: 6–184 months) (Table 2).

**Table 2:** Treatment and behavioral related factors of participants. Bahir Dar, Nov, 2017–April, 2018

Variable	Category	Frequency(N)	Percent (%)
Current TB co- infection	Yes	44	10.2
	No	386	89.8
Treated with TB before	Yes	119	27.7
	No	311	72.3
Addictions	Not addicted	404	94.0
	Smoking	1	0.2
	Alcohol	23	5.3
	Khat	1	0.2
	Smoking, Khat Alcohol	1	0.2
Sexual contacts	only with one person	301	70.0
	More than one partner	116	27.0
	No sex history	13	3.0
Condom use	Yes	123	28.6
	No	307	71.4
Type of start regimen	1a (D4T,3TC,NVP)	63	14.7
	1b (D4T,3TC,EFV)	14	3.3
	1c (AZT,3TC,NVP)	133	30.9
	1d (AZT,3TC,EFV)	34	7.9
	1e (TDF,3TC,EFV)	149	34.7
	1f (TDF,3TC,NVP)	37	8.6
Regimen Changed	No	354	82.3
	Yes	76	17.7
Baseline WHO staging	1	60	14.0
	2	59	13.7
	3	267	62.1
	4	44	10.2
Drug discontinuation history	Yes	90	20.9
	No	340	79.1
Time of drug use	Constant time	185	43.0
	Irregular time	245	57.0
Adherence	Good	333	77.4
	Fair	35	8.2
	Poor	62	14.4
	**Total**	**430**	**100.0**

D4T- Stavudine, 3TC- Lamivudine, AZT-Zidovudine, NVP-Nevirapine, EFV- Efavirenz, TDF-Tenofovir Disoproxil Fumarate

At baseline, 267(62.1%) of the participants were at WHO stage 3. Remarkably, at the time of data collection, 426 (99.1%) of PLWHIV were at WHO stage treatment 1 (T1) showing clinical improvement. Twenty-one percent of the participants have history of treatment interruption and that of 43% disclosed irregularity on medication time. With regard to adherence, 333(77.4 %) of patients were with good (>95%) adherence and 97(22.6%) were with sub-optimal adherence (<95%) (Table 2).

### Prevalence and associated factors of treatment failure

**Figure 1:**
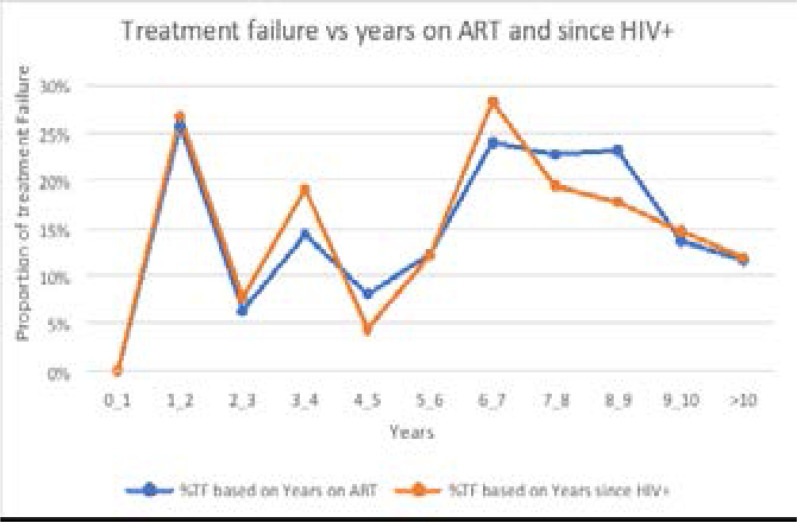
Trends of treatment failure with regard to time since HIV+ and years on ART, Bahir Dar, 2019

Figure 1 below shows that high rate (25%) of TF was reported among people who have a follow up period of 1–2 and 6–7 years. The trends of TF were similar with regard to time since HIV positive and ART follow up period. Taken together, the over all prevalence of VTF with first and second viral load test was gauged at 106 (24.7 %) and 65 (15.1%), respectively. Thus, the utility of second viral load in avoiding premature switching or the re-suppression rate was 41/106 (38.7%).

Closer inspection of the table 3 shows that first line HIV TF was higher among males than female (73.8 % versus 26.2%), among age range of 25–34 years, and among people with primary education 24 (36.9%) than their counterparts (Table 3).

**Table 3:** Logistic regression analysis of factors associated with treatment failure, Bahir Dar, Nov, 2017–April, 2018

Variable		Treatment failure		Total	COR (95%CI), P	AOR (95%CI), P
	Category	Yes, n (%)	No, n (%)	n (%)		
Gender	Female	17 (26.2)	232 (63.6)	263(61.2)	1.00	
	Male	48 (73.8)	133 (36.4)	167(38.8	4.9(2.2–8.9),0.0	2.9(1.45–9)0.002
BMI Category	<18.5	23(35.4)	72(19.7)	95(22.1)	1.00	1.00
	18.6 –25.5	38(58.5)	222(60.8)	260(60.5)	0.5(0.29–0.9)0.04	1.00
	25.6–30.0	4(6.2)	67(18.4)	71(16.5)	0.1(0.06–0.5)0.003	1.00
	>30.0	0.0(0 )0.0	4(0.9)	4(0.9)	1.00	1.00
Marital status	Married	22(33.8)	174(47.7)	196(45.6)	1.1(.4–2.8).7	0.7(0.2–2.1)0.5
	single	15(23.1)	56(15.3)	71(16.5)	1.6(0.8–3.4)0.04	0.7(0.2–3.0)0.6
	Divorced	21(32.3)	72(19.7)	93(21.6)	2.3(1.1–4.4) 0.04	1.0(0.3–3.6)0.9
	Widowed	7(10.8)	63(17.3)	70(16.3)	1.00	
Current TB	Yes	4(6.2)	40(11.0)	44(10.2)	0.5(0.1–1.5)0.2	2.3(0.6–9.3)0.2
	No	61(93.8)	325(89.0)	386(89.8)	1.00	
Treated with TB before	yes	16(24.6)	103(28.2)	119(27.7)	0.8(0.4–1.5)0.5	1.5(0.6–3.4)0.3
	No	49(75.4)	262(71.8)	311(72.3)	1.00	
Sexual behavior	0 or 1 sexual contact	27(41.5)	287(78.6)	314(73.0)	1.00	
	≥ 2 sexual contact	38(58.5)	78(21.4)	116(27.0)	5.1(2.9–9.0) .0	3.2(1.6–6.2)0.0
Condom use habit	yes	13(20.0)	110(30.1)	123(28.6)		
	No	52(80.0)	255(69.9)	307(71.4)	1.7(0.9–3.2)0.09	
Types of baseline regimen	1a	7(10.8)	56(15.3	63(14.7)	1.00	
	1b	2(3.1)	12(3.3)	14(3.3)	1.3(.2–7.2) 0.73	
	1c	27(41.5)	106(29)	133(30.9)	2.0(.8–5.0) 0.11	
	1d	8(12.3)	26(7.1)	34(7.9)	2.4(.8–7.5) 0.11	
	1e	15(23.1)	134(36.7)	149(34.7)	0.8(.3–2.3) 0.82	
	1f	6(9.2)	31(8.5)	37(8.6)	1.5(.4–5.01) 0.46	
Were regimen changed?	No	59(90.8)	295(80.8)	354(82.3)	2.3(.9–5.6)0.06	
	Yes	6(9.2)	70(19.2)	76(17.7)		
Baseline WHO staging	1	7(10.8)	53(14.5)	60(14.0)	1.00	
	2	8(12.3)	51(14.0)	59(13.7)	1.1(0.40–3.5)0.75	
	3	39(60.0)	228(62.5)	267(62.1)	1.2(0.5–3.0)0.55	
	4	11(16.9)	33(9.0)	44(10.2)	2.5(0.8–7.1)0.08	
Baseline CD4 count	<200	46(70.8)	169(46.3)	215(50.0)	2.8(1.5–4.9) .00	2.4(1.0–5.5)0.03
	≥ 200	19(29.2)	196(53.7)	215(50.0)		
Current CD4 Count	<200	20(30.8)	49(13.4)	69(16.0)	2.8(1.5–5.2) .001	4.3(1.8–10.0)0.01
	≥ 200	45(69.2)	316(86.6)	361(84.0)		
Adherence	Good	14(21.5)	319(87.4)	333(77.4)	1.00	
	Fair & Poor	51(78.5)	46(12.6)	97(22.6)	25.2(12.949.2)0.001	16.5(8.2–33.1)0.00
Time of drug use	Irregular time	54(83.1)	131(35.9)	185(43)	8.7(4.4–17.3)0.00	3.7(1.7–8.1)0.001
	Exact time	11(16.9)	234(64.1)	245(57)	1.00	

The mean CD4 value of the participant was 455 (±278) and 267.6 cells/mm3 (+-234.5) at the time of study and baseline, respectively. The mean WBC counts was 6.2 x10^6^ (± 1.9) cells/mm^3^ and that of the mean hemoglobin value was 13.5 g/dl (±2.3). The mean value of SGPT and creatinine were 33.6 IU/l (±25.6) and 0.8 mg/dl (±0.3), respectively. No significant differences were found between treatment failed and none treatment failed groups with regard to complete blood cell count, creatinine value, SGPT and hemoglobin value in the present study.

Together, in the present study, TF is associated with gender, treatment discontinuation habit, irregular time of medication intake, poor adherence, low CD4 value (<200 cells/ml) at current and baseline and sexual behavior.

## Discussion

The study revealed that first line HIV TF was 15.1%. Based on WHO definition of TF, this prevalence report is considered as high 26. Treatment failure could be either due to drug resistance which can occurred before or after start of treatment, super infection with resistant strain 27, 28 or clinical failure. Our result was in line with the studies done in Amhara 16, 17. However, our report was higher than national average of VTF, 5.8%^12^, ^13^, ^18^. Moreover, the present study was also higher than study in the Kenya, 10% 29 with VL cut-off value >1000 RNA copies/ml. Treatment failure was also higher in the first years of their life with HIV and with ART. A study from Addis Ababa using a sensitive allele-specific polymerase chain reaction reported a 6.5% pretreatment drug resistance (TDR) prevalence and two studies from Northwest Ethiopia reported a 3.3% and a 5.6% TDR prevalence, respectively 11

Male gender showed significant association with treatment failure (AOR 3 95% CI 1.5–6, p 0.002). This result was in line with previous studies from Ethiopia, Bale 30 China 20 Uganda 31 and Burkina Faso 32. This difference might be due to behavioral factors such as high rate of alcoholism, smoking, and field work among males that collectively leads to poor adherence 32, drug interruption 33, lost to adhere on time of medication^34^ which collectively leads to TF.

With successive increases in sexual contacts, there was also an increase in TF (AOR 3.79, 95% CI 1.93–7.45, p 0.00). This might be due to exchange of mutated virus and sub types between sexual contacts 27. WHO classify adherence as good (>95%), fair (85–95%) and poor (<85%) 35. Adherence measures the extent to which the patient taking medication appropriately 36. Our study classify adherence based on patients' subjective reports. No verbal report of any medication interruption and drug intake report as per the physician advice is taken as good adherence. Verbal report of medication discontinuation, irregular time of medication accompanied by an overall rational judgment by the patient were considered as either fair or poor adherence. Several lines of evidences including our study support the link between sub-optimal adherence with TF (AOR 16.51, 95%CI 8.23–33.12, p0.00) 37, 38. Poor drug adhererance leads not only TF but also drug resistance. Factors that promote resistance evolution include a high reproductive number, extended drug holidays and poor adherence 39. TB/HIV co-infection and months of treatment did not show association with TF. This finding was corroborated with report in Cameron 40.

Overall, these results indicate that irregular time of medication, drug discontinuation, poor adherence, multiple sexual history, male gender, baseline and current CD4 count<200 were significantly associated with first line HIV TF.

## Limitations

Human immune deficiency virus drug resistance test was not done which could distinguish drug resistance from clinical failure. Moreover, time of enrollment on ART was not accounted for, which poses major concerns in terms of bias in the estimates.

## Conclusion and Recommendations

In general, first line HIV treatment failure was gauged at 15.1% and re-suppression rate after intensive adherence was 38.7%. Male gender, drug holidays, multiple sexual partner, sub-optimal adherence, lower baseline and current CD4 counts were the potential factors responsible for TF. Hence, sustained counseling and advice should be given targeting risk groups such as male, interrupter, people having low CD4 value. To identify the driving factor, follow-up study is desirable.

## Acronyms and abbreviations

3TC: Lamivudine; AIDS : Acquired Immunodeficiency Syndrome; APHI: Amhara Public Health Institute; ART: Antiretroviral Therapy; ARV: Antiretroviral; AZT: Zidovudine (also known as ZDV); BMI: Body Mass Index; CD4+ cell: T-lymphocyte bearing CD4 receptor; CDC: Centers for Disease Control and Prevention; CRISPR: clustered regularly interspaced short palindromic repeat; Cas9: CRISPR-associated nuclease 9 D4T:Stavudine; DM: Diabetes Mellitus; DNA: Deoxyribonucleic Acid; DRV: Darunavir; EFV: Efavirenz; ETV: Etravirine; FHRH: Felege Hiwot Referal Hospital; FMOH: Federal Ministry of Health; FTC: Emtricitabine; HAART: Highly Active Antiretroviral Therapy; HIV: Human Immunodeficiency virus; HIVDR: HIV Drug Resistance; HIVRNA: Human Immuno Deficiency Virus Ribonucleic Acid; IDV: Indinavir; LPV: Lopinavir; LPV/r: Lopinavir/ritonavir; MOH: Ministry of Health; NNRTI: Non-Nucleoside Reverse Transcriptase Inhibitor; NRTI: Nucleos(t)ide Reverse Transcriptase Inhibitor; NVP: Nevirapine; PI: Protease Inhibitor; PLHIV: People Living with HIV; PR: Protease; PVL: Plasma Viral Load; RNA: Ribonucleic Acid; RT: Reverse Transcriptase; RTI: Reverse Transcriptase Inhibitor; SQV: Saquinavir; TB: Tuberculosis; TDF: Tenofovir Disoproxil Fumarate; TF: Treatment Failure; UNAIDS: United Nations Program on HIV/AIDS; VL: Viral Load; WHO: World Health Organization

## Declarations

### Availability of data and material

The datasets supporting the conclusions of this article are included within the article.
